# A Rare Cause of Cushing's Syndrome: an Adrenocorticotropic Hormone (ACTH)-Secreting Pheochromocytoma

**DOI:** 10.7759/cureus.37883

**Published:** 2023-04-20

**Authors:** Maria Leonor Guia Lopes, Carlos Bello, Lucília Carvalho, Clotilde Limbert, João Sequeira Duarte

**Affiliations:** 1 Endocrinology Department, Hospital Egas Moniz, Lisbon, PRT; 2 Endocrinology Department, Hospital da Luz Lisboa, Lisbon, PRT; 3 Pathology Department, Hospital Egas Moniz, Lisbon, PRT

**Keywords:** hypokalemia, adrenal gland, pheochromocytoma, ectopic cushing’s syndrome, cushing’s syndrome

## Abstract

Cushing's syndrome (CS) is a rare clinical entity that results from prolonged exposure to supraphysiological levels of glucocorticoids. It may result from adrenocorticotropic hormone (ACTH)-dependent or nondependent stimuli. In very rare cases, ACTH production does not derive from the pituitary gland but is of an ectopic origin.

We present a case of a 51-year-old woman with cushingoid physical features, who was admitted to the emergency department with a hypertensive crisis, hyperglycemic state, and severe hypokalemia. During the diagnostic workup, the unequivocal confirmation of hypercortisolism status and ACTH elevation led to the suspicion of Cushing’s disease. However, additional testing with a corticotropin-releasing hormone test and inferior petrosal sinus sampling suggested against this etiology. Surprisingly, a body computerized tomography scan incidentally revealed the presence of a left adrenal mass with a high uptake in a ^68^Ga-DOTANOC positron emission tomography scan. The further investigation documented elevated urinary metanephrines and normetanephrines. The patient was referred for surgical resection of the adrenal gland, and the anatomopathological report revealed the diagnosis of ACTH-secreting pheochromocytoma without local invasion or malignant features. Diabetes mellitus, hypertension, hypokalemia, and cushingoid stigmata were remitted soon after surgery.

ACTH-secreting pheochromocytomas are extremely rare causes of CS. This diagnosis demands a high level of clinical suspicion and should be equated in the presence of severe metabolic changes overlapping CS's physical features. The total reversal of metabolic and clinical symptoms after surgical resection highlights the need to remember this etiology when performing a CS workup.

## Introduction

Cushing’s syndrome (CS) is a rare endocrine disorder with high morbidity and mortality rates. Although the most frequent cause of CS is chronic treatment with glucocorticoids, pituitary adrenocorticotropic hormone (ACTH)-producing adenomas may be the culprit of hypercortisolism in some cases. This CS cause is usually more prevalent in women and less common in the pediatric population [1-3]. In extremely rare scenarios, ectopic ACTH production may result in CS called ectopic CS (ECS). In those cases, metabolic conditions, such as hyperglycemia, hyperlipidemia, and hypokalemia, are commonly severe in addition to the usual cushingoid features. Moreover, in ECS, cushingoid physical manifestations are not so prompt to be recognized, considering the abrupt and severe onset of hypercortisolism [4].

The most frequent cause of ECS is an abnormal expression of ACTH receptors in pulmonary malignancies, such as bronchial carcinoma and gastrointestinal neuroendocrine tumors [1-3,5,6]. In extremely rare cases, other neuroendocrine tumors, namely pheochromocytomas, can ectopically produce ACTH, leading to an unregulated glucocorticoid production and resulting in the development of CS.

We report an extraordinary case of a patient presenting with severe symptoms of simultaneous hypercortisolism and catecholamine excess caused by an ACTH-producing pheochromocytoma.

## Case presentation

The authors report the case of a 51-year-old woman with a medical history of hypertension, tobacco use, and a recent diagnosis of dyslipidemia and oral candidiasis. She was admitted to the emergency department (ED) with a hypertensive crisis and hyperglycemic state. The patient reported sequential onset of multisystemic complaints within the previous six months, describing progressively increasing fatigue, depressive symptoms, and changes in her usual appearance. She also reported a higher arterial pressure profile despite her usual antihypertensive treatment.

At the admission, the patient presented with sunken eyes, pale mucous membranes, periorbital edema, moon facies, alopecia, hirsutism, petechiae lesions dispersed over both upper limbs, skin hyperpigmentation, increased abdominal circumference (104 cm), and proximal myopathy. Ophthalmological observation revealed the presence of bilateral flare retinal hemorrhages, probably associated with hypertension.

Urgent blood workup showed severe hypokalemia (2.1 mmol/L) as well as hyperglycemia (421 mg/dL) (Table 1).

**Table 1:** Laboratory data at the admission to the emergency department

Laboratory test	Result	Reference range
Hemoglobin (g/dL)	14.6	12.0-15.0
Mean corpuscular volume (fL)	94.6	80.0-96.1
White blood cells (×10^9^/L)	13.6	4.0-10.0
Platelets (10^9^/L)	137	150-400
Sodium (mmol/L)	143	136-145
Potassium (mmol/L)	2.1	3.5-5.1
Phosphate (mg/dL)	2.9	2.5-4.5
Magnesium (mg/dL)	2.4	1.7-2.2
Calcium (mg/dL)	9.6	8.6-10.0
Albumin (g/L)	4.1	3.5-5.2
Creatinine (mg/dL)	0.8	0.5-0.9
Glomerular filtration rate (mL/min/1.73 m^2^)	96	>60
Glucose (mg/dL)	421	74-110
Hemoglobin A1c (%)	10.5	<5.7%
Triglycerides (mg/dL)	146	<150
Total cholesterol (mg/dL)	205	<200
High-density lipoprotein cholesterol (mg/dL)	44	>45
Low-density lipoprotein cholesterol (mg/dL)	132	<116

Considering the severity of the clinical presentation, the patient was admitted to the endocrinology department. The following workup allowed the authors to unequivocally confirm the diagnosis of endogenous hypercortisolism considering the results obtained from a 24-hour urinary free cortisol measurement and an overnight 1 mg dexamethasone suppression test (both 20-fold above the upper limit of normal). Moreover, the patient presented with cortisol rhythm reversal and serum ACTH elevation (Table 2).

**Table 2:** Laboratory data at the endocrinology department ACTH, adrenocorticotropic hormone

Laboratory test	Result	Reference range
Morning (8 a.m.) cortisol after 1 mg dexamethasone suppression test (μg/dL)	49	<1.8
24-hour urinary cortisol (μg/24 h)	1268.1	8.0-63.0
Morning (8 a.m.) cortisol (μg/dL)/late night cortisol (11 p.m.) (μg/dL)	49.8/52.5	6.2-19.4
Morning (8 a.m.) ACTH (pg/mL)	94.5	7.2-63.3
Prolactin (ng/mL)	13.9	4.8-23.3
Follicle-stimulating hormone (U/L)	30.6	25.8-135 (postmenopausal)
Luteinizing hormone (U/L)	14.4	7.7-59 (postmenopausal)
Estradiol (pg/mL)	6.77	<138 (postmenopausal)
Thyroid-stimulating hormone (μUI/mL)	0.29	0.270-4.200
Free thyroxine (T4) (pmol/L)	19.3	12.0-22.0
Aldosterone (pg/mL)	36.0	10-160
Plasmatic renin activity (ng/mL/h)	0.7	0.2-1.6
Total testosterone (ng/dL)	7.6	2.9-40.8
17-Hydroxyprogesterone (ng/mL)	0.8	<2.8
Dehydroepiandrosterone sulfate (μg/dL)	41	35-256
Chromogranin A (nmol/L)	10.4	<6
Urinary fractionated metanephrines (μg/24 h) - first sample	405	<276
Urinary fractionated normetanephrines (μg/24 h) - first sample	1429	<426
Urinary fractionated metanephrines (μg/24 h) - second sample	529	<276
Urinary fractionated normetanephrines (μg/24 h) - second sample	2163	<426

It is worth noting that after taking the 1 mg of dexamethasone, the patient developed psychotic symptoms, requiring mood stabilizing therapy. This rare complication is also an important surrogate of the severity of the patient’s endogenous hypercortisolism and has led to the eviction of dexamethasone suppression tests in the subsequent investigation.

**Figure 1:**
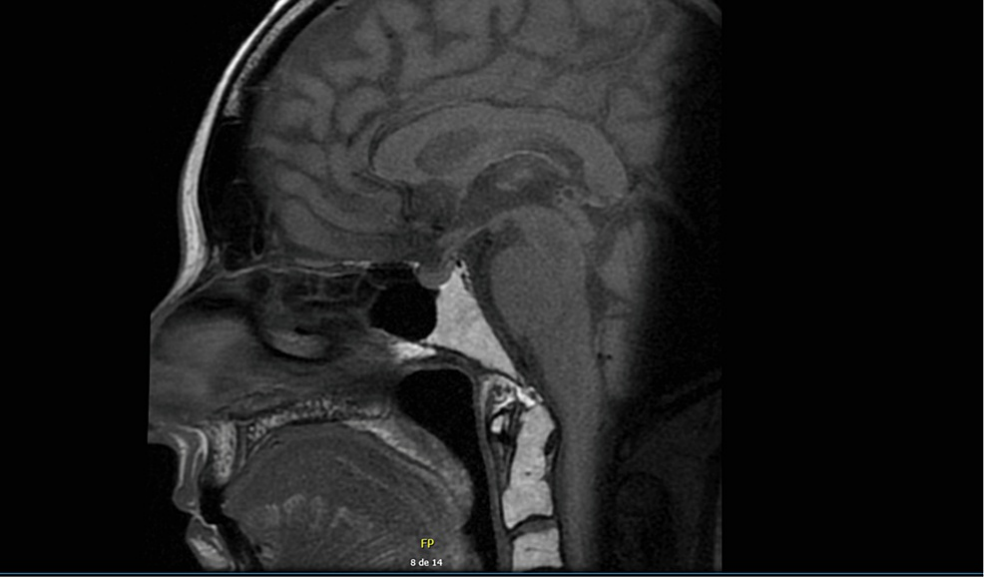
Sagittal view of pituitary MRI (T1-weighted) MRI, magnetic resonance imaging

Cranial magnetic resonance imaging (MRI) showed a slight asymmetry of the parasellar region’s floor, with a discrete pituitary stem bulging to the left. These findings suggested the possibility of Cushing’s disease (CD) (Figure 1). A bilateral inferior petrosal sinus sampling was performed without success due to a technically difficult catheterization. Therefore, a corticotropin-releasing hormone stimulation test (bovine version) was conducted, and the results formally excluded a pituitary origin of hypercortisolism.

**Figure 2:**
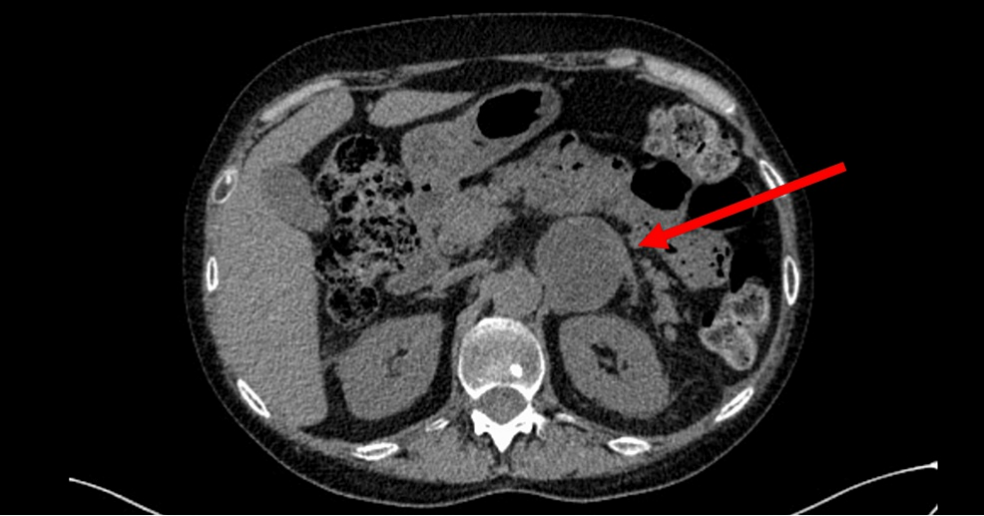
Abdominal CT revealing a 44 × 40 mm left adrenal mass (red arrow) CT, computerized tomography

An ECS was then considered, and a full-body computerized tomography (CT) scan was performed. It excluded the presence of suspicious lung masses, but it unexpectedly revealed a space-occupying lesion in the left adrenal gland with 44 × 40 mm, contrast-enhancing, without late phase washout, and no apparent local lymphovascular invasion (Figure 2). The positron emission tomography with ^68^Ga-DOTANOC showed a predominantly peripheral somatostatin receptor expression in the mass confirming its neuroendocrine nature.

Additional tests revealed catecholamine excess with an elevation of urinary fractionated metanephrines and normetanephrines in two different measurements and a confirmatory positive clonidine suppression test (Table 2). Given the diagnosis of pheochromocytoma, a multidisciplinary team discussed the case, and the patient was referred to surgical adrenal resection. In the preoperative period, she underwent IV potassium therapy, intensive insulin therapy, trimethoprim-sulfamethoxazole (TMP-SMX) prophylaxis, and alpha- and beta-adrenergic blockade.

**Figure 3:**
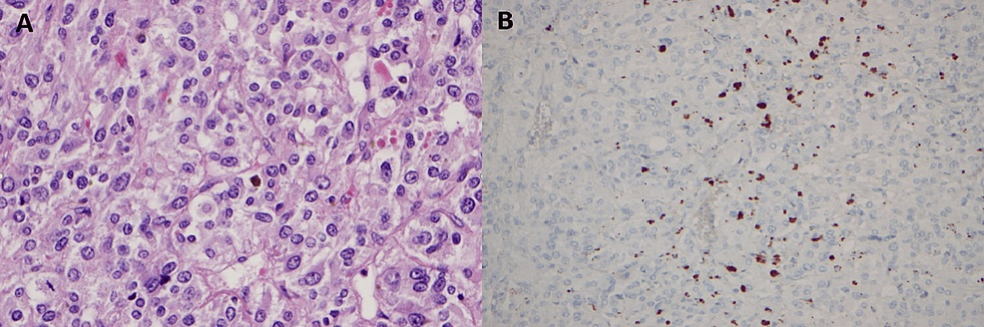
Microscopy showing a classical appearance of pheochromocytoma - large cells with granular amphophilic cytoplasm and mitosis A: Hematoxylin and eosin staining B: ACTH expression by immunohistochemistry ACTH, adrenocorticotropic hormone

A left laparoscopic adrenalectomy was performed. The pathology report revealed the presence of pheochromocytoma with ACTH immunopositive staining. No capsular or vascular invasion was determined, and Ki67% expression was absent in the tumor cells, showing a low proliferative activity. The mass accounted for a total score of zero in the Grading System for Adrenal Pheochromocytoma and Paraganglioma Score (Figure 3).

In the postoperative period, the patient started prophylactic anticoagulation with enoxaparin (during six weeks) and glucocorticoid replacement (10-12 mg/m2/day of hydrocortisone) and continued prophylactic TMP-SMX antibiotic therapy for two weeks, following the most recent recommendations in CS management [7]. Analytical re-evaluation was performed one week postoperatively, revealing the patient’s hypercortisolism remission. Fractionated urinary metanephrines measurement was also normal four weeks after surgery (Table 3).

**Table 3:** Analytical profile after surgical resection of the ACTH-producing pheochromocytoma ACTH, adrenocorticotropic hormone

Laboratory test	Result	Reference range
Morning (8 a.m.) cortisol (μg/dL)	1.8	6.2-22
Urinary fractionated metanephrines (μg/24 h)	85	<276
Urinary fractionated normetanephrines (μg/24 h)	355	<426

One year after surgical treatment, the patient remained clinically stable with no evidence of disease recurrence and total reverse of hypokalemia (potassium serum level, 3.8 mmol/L), diabetes mellitus (hemoglobin A1c, 5.2%), hypertension, and cushingoid physical signs. Genetic testing was negative for hereditary causes of pheochromocytoma. Life-long annual catecholamines and serum cortisol determinations were recommended.

## Discussion

The authors present an extraordinary clinical case of CS secondary to ectopic production of ACTH by a pheochromocytoma. Apart from the rarity of this etiology, some particularities also made this case so unusual and are worth discussing.

ECS is most commonly caused by bronchial carcinomas and non-small cell carcinomas of the lung. Given the patient’s smoking history, the hypothesis of malignant neoplasm of the lung seemed the most probable. However, the unusual finding of an adrenal pheochromocytoma as the source of the patient’s hypercortisolism contradicted all expectations.

ACTH-producing pheochromocytomas are extremely rare, with less than 100 cases reported in the literature [8]. These tumors present as clinical challenges since their initial manifestations markedly differ from the classical clinical syndrome with headache, diaphoresis, hypertension, and tachycardia. CS symptomatology caused by cortisol overproduction frequently remains the only diagnostic clue in these patients [9]. These cases are often characterized by an extremely rapid onset of hypercortisolism leading to a swift development of cushingoid clinical signs (hyperandrogenism, proximal myopathy, and skin hyperpigmentation). As an ECS cause, they may also present with severe metabolic disturbances with hypokalemia, hyperglycemia, and hypertension.

In this case, the patient was admitted to the ED with a hypertensive crisis and severe metabolic and electrolyte dysfunction requiring immediate care. Hypokalemia is one of CS's most prevalent and specific metabolic features, especially in ECS (occurs in 70-95% of ECS vs 10% in CD). In these cases, hypokalemia results from the mineralocorticoid action of the glucocorticoid excess. Frequently, ECS produces higher levels of hypercortisolism than CD, resulting in more severe hypokalemia [1-3,10].

On the other hand, the fact that the patient presented with a psychotic outbreak after the 1 mg dexamethasone suppression test is also an important clinical warning that must be taken into consideration. Using exogenous glucocorticoids can lead to severe psychiatric decompensation, especially in individuals with underlying psychiatric pathology. In CS, the cumulative glucocorticoid overload further enhances the probability of this exacerbation. This iatrogenic possibility may be considered during the dexamethasone suppression tests in daily practice [11].

Finally, evidence suggests that hypercortisolism may markedly predispose to hypercoagulability with thrombotic and thromboembolic events and general and opportunistic infections [7,12-15]. The most recent expert recommendations consider that these patients present a higher thrombotic risk and that thromboprophylaxis benefits might outweigh the risks, especially in cases of clinically severe CS. Thromboprophylaxis could even be reasonable to be extended up to two to three months after surgical treatment. Our patient started on prophylactic anticoagulation as described above [7,11-14].

Considering the patient's glucocorticoid relative immunosuppression, the expert recommendations also consider that *Pneumocystis jirovecii* prophylaxis should be used for all patients with ectopic or severe cases of CS. TMP-SMX antibiotic therapy was maintained in our patient for two weeks after surgery [7,15].

In this particular case, considering the tumor's dimension and the patient’s performance status, surgical resection remained the best treatment option. However, in some cases, adrenal surgery might represent a life-risking procedure and conservative treatment approaches should be considered. Medical treatment with steroidogenesis inhibitors and control of comorbidities might represent a possible solution. Moreover, adrenal artery embolization might be considered to decrease blood flow to the tumor and reduce its hormonal production [3].

## Conclusions

The extremely rare possibility of a pheochromocytoma-producing ACTH should be equated in CS, especially when metabolic changes overlap the physical features and pulmonary and other neuroendocrine tumors are excluded. All clinicians should be alert to this clinical scenario, given its high morbidity and mortality rates and its possible cure through surgery.

In patients with a previous medical history of psychiatric disease, careful use of dexamethasone suppression tests is recommended since they might lead to psychiatric decompensation. The therapeutic management of these patients remains difficult, and it urges the development of evidence-based guidelines to improve the quality of care.
